# Quasiparticle interference and nonsymmorphic effect on a floating band surface state of ZrSiSe

**DOI:** 10.1038/s41467-018-06661-9

**Published:** 2018-10-08

**Authors:** Zhen Zhu, Tay-Rong Chang, Cheng-Yi Huang, Haiyang Pan, Xiao-Ang Nie, Xin-Zhe Wang, Zhe-Ting Jin, Su-Yang Xu, Shin-Ming Huang, Dan-Dan Guan, Shiyong Wang, Yao-Yi Li, Canhua Liu, Dong Qian, Wei Ku, Fengqi Song, Hsin Lin, Hao Zheng, Jin-Feng Jia

**Affiliations:** 1 School of Physics and Astronomy, Shanghai Jiao Tong University, Shanghai, 200240 China; 2 Department of Physics, National Cheng Kung University, Tainan, 701 Taiwan; 3 Institute of Physics, Academia Sinica, Taipei City, 11529 Taiwan; 4 College of Physics, Nanjing University, Nanjing, 210093 China; 5 Department of Physics, Massachusetts Institute of Technology, Cambridge, MA 02139 USA; 6 Department of Physics, National Sun Yat-Sen University, Kaohsiung, 80424 Taiwan; 7 Collaborative Innovation Center of Advanced Microstructures, Nanjing University, Nanjing, 210093 China

## Abstract

Non-symmorphic crystals are generating great interest as they are commonly found in quantum materials, like iron-based superconductors, heavy-fermion compounds, and topological semimetals. A new type of surface state, a floating band, was recently discovered in the nodal-line semimetal ZrSiSe, but also exists in many non-symmorphic crystals. Little is known about its physical properties. Here, we employ scanning tunneling microscopy to measure the quasiparticle interference of the floating band state on ZrSiSe (001) surface and discover rotational symmetry breaking interference, healing effect and half-missing-type anomalous Umklapp scattering. Using simulation and theoretical analysis we establish that the phenomena are characteristic properties of a floating band surface state. Moreover, we uncover that the half-missing Umklapp process is derived from the glide mirror symmetry, thus identify a non-symmorphic effect on quasiparticle interferences. Our results may pave a way towards potential new applications of nanoelectronics.

## Abstract (web summary)

A new type of surface state has recently been reported in ZrSiSe but its physical properties remain unclear. Here, Zhu, Chang, Huang, et al. report rotational symmetry breaking, healing effect and anomalous Umklapp scattering arising from the nonsymmorphic effect of the floating band state on ZrSiSe (001) surface.

## Introduction

Research into surface states has been conducted for several decades and has recently begun to flourish again due to the discovery of topologically non-trivial materials^1,2^. Several topological surface states have been uncovered with prominent examples including the spin-momentum locked Dirac cones in topological insulators^3,4^ and the disconnected Fermi arcs in Weyl semimetals^5,6^_._ Many characteristic phenomena, e.g., the prohibition of electron back scattering on a topological insulator surface^7,8^, the tunable mass acquisition of surface fermions in a topological crystalline insulator^9^ and the electronic sink effect in a Weyl semimetal^10–13^ have been discerned through quasiparticle interference (QPI) approaches. These have all been proven advances in the understanding of the unconventional two-dimensional electron gases. Therefore, the search for new classes of surface states with intriguing physical consequences is an invaluable endeavor in condensed matter physics.

**Fig. 1:**
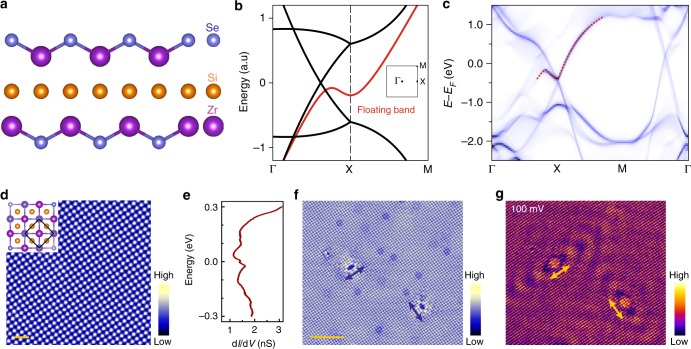
Structural and electronic properties of ZrSiSe. **a** Crystal structure of ZrSiSe, which features a non-symmorphic *P*4/*nmm* space group. The Si layer serves as a glide mirror plane $\left( {\left. {M_z} \right|\frac{1}{2}\frac{1}{2}0} \right)$. The weak Van der Waals interaction between adjacent Se–Zr–Si–Zr–Se quintuple layers provides a natural cleaving surface between Se surfaces [(001) surface]. Blue, yellow, purple balls stand for Se, Si, Zr atoms, respectively. **b** Sketch of band structure without taking spin–orbit coupling into account. Nodal line bulk state and floating band surface state are plotted in black and red respectively. The non-symmorphic symmetry in ZrSiSe protects the Dirac nodes located at the X point, as well as generates an unconventional type of floating band surface state, on the (001) surface. Inset is the surface Brillouin zone (BZ) with high symmetry points marked. **c** Calculated surface band structure of ZrSiSe(001). The floating band state is highlighted in red. **d** STM image (0.1 V, 0.2 nA) demonstrating the atomic lattice on ZrSiSe(001) surface. The lattice constant is measured to be 0.37 nm. Both the inset crystal structure and the STM image show that the surface preserves *C*_4*v*_ symmetry. Scale bar stands for 1 nm. **e** Typical d*I*/d*V* spectrum measured on top of a Se atom in a defect free region. **f** STM image (300 mV, 1 nA) showing a large-scale morphology. Arrows indicate two defects, which apparently break *C*_4*v*_ symmetry. Scale bar stands for 5 nm. **g** d*I*/d*V* map acquired at same region as **f**. *C*_2*v*_ symmetric standing wave patterns around each defect are clearly discerned

ZrSiSe is a newly discovered non-symmorphic topological Dirac nodal-line semimetal^14–19^ and is part of the class of materials which includes ZrSiS, ZrSiSe, and ZrSiTe. Its bulk band features a linear dispersion in the energy range as broad as 2 eV, much larger than other known Dirac materials and presents the ZrSiSe class of materials as an ideal candidate to target new related physics^17–19^. Indeed, a high electron mobility and a butterfly magnetoresistance was discovered by transport measurements^20^. More importantly, a very recent study revealed an unconventional floating band surface state on ZrSiS but which is also applicable to ZrSiSe and ZrSiTe. Its origin is directly derived from the non-symmorphic symmetry of the crystal and is distinct from the well-known Shockley type or dangling-bond type surface state^21^. As demonstrated in Fig. 1a, ZrSiSe is a layered material and crystallizes into a tetragonal lattice with a space group *P*4/*nmm* (#129), which is shared with a broad variety of quantum materials, e.g., nematic Fe-based superconductor NaFeAs^22,23^ and heavy-fermion compound with antiferromagnetism (AFM) CeRuSiH_1.0_^24^. In the electronic band structure of ZrSiSe (Fig. 1b), non-symmorphic symmetry enforces the bulk bands to be doubly (quadruply if considering spin degrees of freedom) degenerate along entire X-M line; in other words, there exists a Dirac nodal line on the Brillouin Zone (BZ) boundary. On its (001) surface, the symmetry breaking splits a two-dimensional electronic state from the bulk Dirac band, termed as a floating band. Figure 1c presents the first principle calculation result where the floating band is highlighted. Obviously, this previously unknown surface state exists in a wide range of *P*4/*nmm* symmetric crystals which goes beyond topologically non-trivial materials. However, other than the identification of its origin, little is known about this surface state.

Among all surface sensitive measurements, QPI which is acquired via scanning tunneling microscopy (STM) may be the most direct method to reveal the unique physics of surface states. An ordinary QPI map measures surface standing wave induced by a number of (usually various types of) point defects. However, the local geometrical and chemical structures of different types of defects carry distinctive information.

Here, we apply a single-defect induced QPI (s-QPI) approach, which is of both experimental and theoretical challenge, to directly measure the interferences at both single Si-defect and Zr-defect sites on the ZrSiSe (001) surface. In addition to the previously insightful QPI discoveries on ZrSiS^25,26^ and ZrSiSe^27^, we directly identify the characteristic properties of a floating band surface state. Moreover, our theoretical analysis reveals the observed anomalous Umklapp process to in principle exist in a broad class of non-symmorphic crystals.

## Results

### Rotational symmetry breaking feature

An overview of our low-temperature STM images, spectroscopy and d*I*/d*V* map on a ZrSiSe (001) surface is shown in Fig. 1. The atomically resolved STM image in Fig. 1d clearly shows the square lattice of our high quality ZrSiSe sample, in which the *C*_4*v*_ symmetry and the measured lattice constant of 0.37 nm confirms the cleaved surface to be the (001) orientation. The measured local density of state from the d*I*/d*V* spectrum (Fig. 1e) exhibits non-vanishing intensity at the Fermi level, revealing the (semi-)metallic nature of our sample. Interestingly, our STM image (Fig. 1f) and d*I*/d*V* map (Fig. 1g) acquired at an energy near the Fermi level demonstrates an unusual ripple pattern. The pattern contains two orthogonal features, each clearly breaking the *C*_4*v*_ symmetry of the crystal surface, and which were not observed on d*I*/d*V* maps measured on a cousin material ZrSiS^25,26^.

### Healing effect

**Fig. 2:**
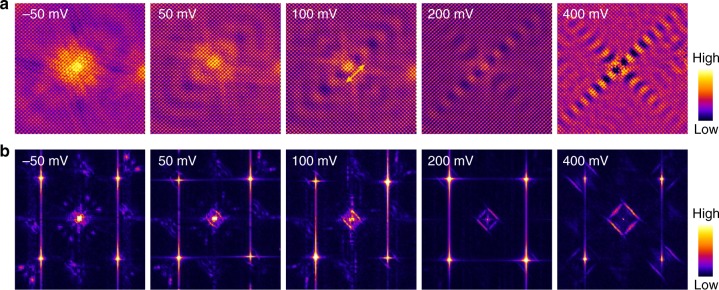
*C*_2*v*_ symmetric interferences on ZrSiSe(001) in single-defect-induced quasiparticle interference (s-QPI) patterns. **a** and **b** are voltage-dependent d*I*/d*V* maps (18×18 nm^2^, 400 mV, 1 nA) and Fourier transformed (FT) d*I*/d*V* maps, depicting the real and reciprocal space s-QPI patterns arising from a single *C*_2*v*_ symmetric defect, respectively. The defect is attributed to a Si vacancy. With increasing bias voltage, the intrinsic *C*_4*v*_ symmetry gradually recovers. The arrow indicates the wave propagating direction

In order to reveal the unique properties of the floating band surface state, we performed systematic s-QPI measurements on ZrSiSe(001). Three characteristics stand out in the voltage-dependent d*I*/d*V* maps and their corresponding fast Fourier transforms (FFTs). First, in contrast to the *C*_4*v*_ pattern expected from the crystalline symmetry, defects showing *C*_2*v*_ symmetric pattern are also found. Figure 2a shows a clear standing wave pattern around the point defect located at the center of the image in the voltage range starting from −50 mV. The wavelength shrinks with elevated bias voltage, thus proving that the surface quasiparticle possesses an electron like band. Clearly, from the map taken at an energy close to Fermi level, i.e., 50 mV, the wave only propagates along one direction. Second, from both Fig. 2a, b, one can discern that the rotational symmetry breaking phenomenon gradually disappears at a higher bias around 400 meV, indicating a healing effect occurring in the sample.

### Anomalous Umklapp process

**Fig. 3:**
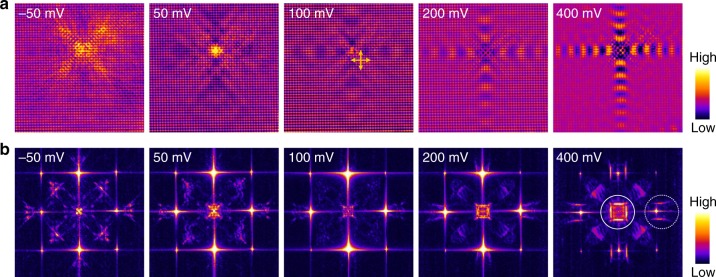
*C*_4*v*_ symmetric s-QPI patterns on ZrSiSe (001). **a** and **b** are voltage-dependent d*I*/d*V* maps (18×18 nm^2^, 400 mV, 1 nA) and FT-d*I*/d*V* maps arising from a single *C*_4*v*_ defect, respectively. The arrows represent the two wave propagating directions. The defect is attributed to a Zr vacancy. Note the scanning directions of all images are rotated *π*/4 with respect to the images in Fig. 2a for technical reasons. The solid (dotted) circle surrounds the QPI feature induced from normal (Umklapp) process

Third, figure 3 shows the expected *C*_4*v*_ symmetric s-QPI patterns (see Supplementary Fig. 1 for STM images of such defects) in which the standing waves propagate equally along two orthogonal directions. Unexpectedly, from the FFT maps in Fig. 3b, one can note that the QPI features around a Bragg point (inside the dotted circle) do not resemble the central pockets (solid circle). This appears to violate the ordinary theoretical understanding of Umklapp scattering and indicates an anomalous structure in the Umklapp process.

## Discussion

It happens that the non-symmorphic symmetry and the special crystal structure of the ZrSiSe class of materials are what lead to the observed QPI features. One unique feature in the structure of its *P*4/*nmm* lattice is the layer dependent location of the rotational axis. The ZrSiSe crystal is formed by alternatively stacked Se–Zr–Si–Zr–Se atomic layers. Each layer itself comprises of a square mesh of atoms which preserves global *C*_4*v*_ symmetry. However, the Si layer forms the glide mirror plane, and thus loses *C*_4*v*_ symmetry locally at each Si atom site in order to fulfill the $\left( {\left. {M_z} \right|\frac{1}{2}\frac{1}{2}0} \right)$ operation. The bulk symmetry gives rise to a (001) surface atomic structure (Fig. 1d) where the Se atoms are located at the corners of the square surface unit cell, the Zr atom sits at the face center, and the Si atoms occupy the edge centers. This naturally leads to the appearance of two inequivalent Si positions, each exhibiting only hidden local *C*_2*v*_ symmetry. In addition to single Si defect, multiple *C*_2*v*_ symmetric Si defects may arrange into multi-directional (Fig. 1g) or uni-directional (Supplementary Fig. 2) configurations. The latter case, if artificially controllable, will give rise to anisotropic scattering of electrons at the Fermi level and consequently lead to a two-fold resistance in ZrSiSe based nanostructures.

**Fig. 4:**
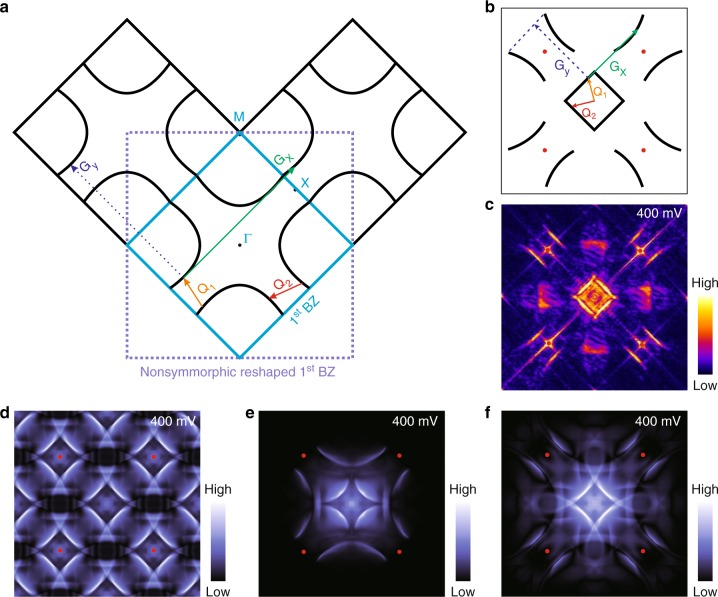
Non-symmorphic effect on a floating band surface state. **a** Schematics depicting the anomalous Umklapp process derived from the non-symmorphic *P*4/*nmm* group. The blue square surrounds the first surface BZ of ZrSiSe(001), in which only the floating band surface state contours are presented. **Q**_**1**_ and **Q**_**2**_ label two dominate scattering vectors. **G**_**x**_ and **G**_**y**_ represent the reciprocal unit vectors. Normal scattering (Q_1_) and Umklapp scatterings (**Q**_**1**_ + **G**_**x**_, and **Q**_**1**_ + **G**_**y**_) are expected to generate the same shapes of QPI patterns in a conventional system with *C*_4*v*_ symmetry. **b** Sketch (not to scale) highlighting the QPI features which arising only from the floating band. The artificially added red dots in **b**, **d**–**f** mark Bragg points. The vectors **Q**_**1**_, **Q**_**2**_, **G**_**x**_, and **G**_**y**_ are defined the same way and in the same directions as in **a**, but with different lengths. The central square (denoted by **Q**_**1**_ and **Q**_**2**_) originates from normal scatterings, while the double arcs near Bragg points are induced by Umklapp scatterings. Note the feature at **Q**_**1**_ **+** **G**_**y**_ is absent, which leads to the half-missing anomalous Umklapp process. **c** The measured *C*_4*v*_ s-QPI pattern at 400 meV. **d** (**e**) is the simulated s-QPI pattern derived from a Zr vacancy by allowing (forbidding) inter-BZ scatterings without considering the non-symmorphic effect. **f** is same as **e** but with considering the non-symmorphic effect. From this, it appears that only **f** reproduces **c**, especially the half-missing Umklapp process. The $\left( {\left. {M_z} \right|\frac{1}{2}\frac{1}{2}0} \right)$ symmetry leads to an extension of the first BZ (purple dotted square in **a**). This non-symmorphic effect naturally induces the half-missing Umklapp interference

In contrast to the crystal structure analysis, we find that first principle simulations and theoretical analysis are crucial to interpret the healing effect occurring in the sample restoring to *C*_4*v*_ symmetry and the anomalous Umklapp process. We begin the discussion by considering s-QPIs on a Zr defect. Having carefully identified the floating band from the other states (see details in Supplementary Fig. 3), we can now draw a schematic constant energy contour (CEC) which only exhibits such bands (Fig. 4a). The floating band pockets manifest as four large rings enclosing the corners of the first BZ. Near a X point, two floating band contours exist approximately parallel to each other, which gives rise to a significantly enhanced nesting vector, i.e., **Q**_**1**_ in Fig. 4a. **Q**_**1**_ and its *C*_4*v*_ rotational partner **Q**_**2**_ together constitute the bright central square in the s-QPI pattern, which is shown in Fig. 4b. In addition to these intra-first BZ scatterings (normal processes), inter-BZ scatterings (Umklapp processes) usually also contribute to the QPI. For example, a normal scattering vector **Q**_**1**_ followed by a unit reciprocal vector (**G**_**x**_ or **G**_**y**_) produces a typical Umklapp process, which should preserve the crystal symmetry. The *C*_4*v*_ point group in ZrSiSe should in principle result in the vector **Q**_**1**_ + **G**_**x**_ or **Q**_**1**_ + **G**_**y**_ generating a QPI contour with the exact shape as **Q**_**1**_, which appears as replica squares at the four Bragg points as shown in Fig. 4d. However, our observation clearly contradicts this ordinary Umklapp process. Concretely, the QPI feature near a Bragg point manifests as a double-parallel arc (Fig. 4b, c) rather than a square. We name this phenomenon, not previously understood, to be an anomalous half-missing Umklapp process, as exactly half of the expected Umklapp pattern (a square) is absent in the observation.

We carry out a T-matrix based Green’s function approach to simulate the s-QPI patterns of a single Zr defect based on three different assumptions. We first consider a band structure simulation without considering any band unfolding or form factor effects. Figure 4d, e show such s-QPI patterns which respectively allow and forbid inter-BZ scatterings. While both results are able to capture the square-shaped central feature, they both fail to reproduce the half-missing patterns around the Bragg points. In contrast in Fig. 4f, by including a sublattice induced form factor effect and forbidding inter-BZ scatterings, we are able to successfully and quantitively simulate the entire s-QPI pattern, and, in particular, the double arcs around Bragg points.

Based on the above analysis, we now understand that the origin of these anomalous half-missing Umklapp processes is actually a direct consequence of the non-symmorphic effect on the energy band structure in a *P*4/*nmm* crystal. In fact, many important effects induced by glide mirror symmetries have been established based on Fe-based superconductors research^22,23^, but the conclusions also apply to the all materials which contain $\left( {\left. {M_z} \right|\frac{1}{2}\frac{1}{2}0} \right)$. We invoke the relevant discovery here to interpret the half-missing Umklapp scattering. Namely, the glide mirror symmetry $\left( {\left. {M_z} \right|\frac{1}{2}\frac{1}{2}0} \right)$ splits the lattice into two sublattices, where the A and B sublattices are glide mirror partners to each other. This induces a particular type of non-trivial form factor, which must be accounted for in a first principle band calculation. Atomic orbitals can be divided into even or odd parity under the mirror *M*_*z*_ operation. By adding a minus sign (a form factor) to odd orbitals on B sublattice but keep all other orbitals intact, the *M*_*z*_ is effectively absorbed by the wavefunctions. In the new wavefunction basis, the fractal translation $\left( {\frac{1}{2}\frac{1}{2}0} \right)$ becomes a good symmetry, which effectively reduces the original unit cell by half and thus expands the area of the first BZ by exactly two folds. In the reconstructed first BZ (dashed line in Fig. 4a), **Q**_**1**_ + **G**_**x**_ changes from an Umklapp to a normal process, which has no reason to be weak or absent. On the other side, **Q**_**1**_ + **G**_**y**_ is now terminated out of the new first BZ and becomes a real inter-BZ scattering, i.e., a real Umklapp process. The suppression or absence of this feature exactly leads to the half-missing Umklapp feature. More profoundly, it indicates that the floating band surface state contains fewer atomic-scale ripples in contrast to the behavior from a dangling-bond derived surface state. The floating band state is thus believed to be weakly bounded to the surface, analogous to a Fermi-arc surface state on a Weyl semimetal^28^.

**Fig. 5:**
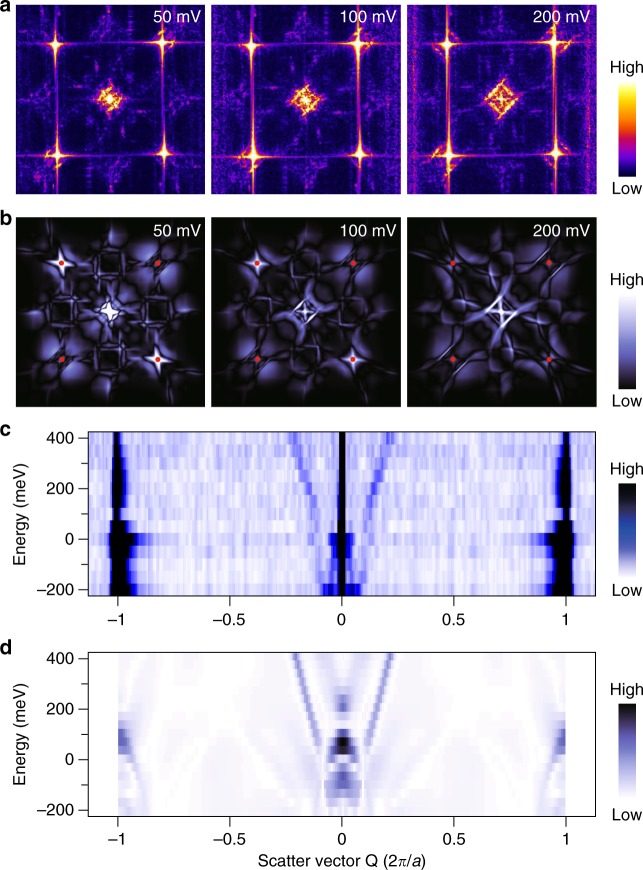
Healing effect on a floating band surface state. **a**, **b** The experimental and simulated QPI patterns from Si defects respectively. The patterns show reduced QPI features compared to Fig. 4. Only a subset of **Q**_**1**_, **Q**_**1**_ + **G**_**x**_ pockets are prominent, while **Q**_**2**_ counterparts are suppressed by the anisotropic defect potential. An energy dependent healing effect of the *C*_4*v*_ breaking is captured by the simulations. **c**, **d** The energy-scattering vector dispersions from experiment and calculation, respectively. The dispersions are taken along the diagonal line in **a** and **b**. From the comparison between **a** and **b**, **c** and **d**, we note that the simulations fit well to the measurements in a large energy range by considering the non-symmorphic effect

By introducing the glide mirror symmetry $\left( {\left. {M_z} \right|\frac{1}{2}\frac{1}{2}0} \right)$ enforced particular type of form factor into our energy band simulations, we can now reproduce the measurements on both *C*_4*v*_ and *C*_2*v*_ types of defects by considering different T-matrices which are directly derived from the first principle simulations on a single defect. In Fig. 5a, we present a set of voltage-dependent experimental *C*_2*v*_ symmetric QPI patterns around uni-directional Si defects (Supplementary Fig. 2). The adjacent panels in Fig. 5b show the simulated patterns which also reproduce the healing effect. The line cuts in Fig. 5c (experimental) and Fig. 5d (theoretical) demonstrate that our simulation corroborates the measurement across a wide energy range, thus proving the robustness of our theory. The healing effect can be understood by analyzing the scattering channels. Namely, at a Si-defect, both a direct scattering channel between Si-*p* orbits and an indirect scattering channel through *p*-*d* orbits coupling (Zr–Si interaction) coexist. Near the Fermi level, the major signal in a *C*_2*v*_ s-QPI pattern is dominated by the direct scattering. However, away from the Fermi level, the signal from Zr-*d* orbits, which carry strong *C*_4*v*_ symmetry, becomes enhanced (see Supplementary Figs. 5–7 for details), and the interference begins to appear less *C*_2*v*_ symmetric. In principle, this healing effect could exist in the entire ZrSiSe-family of topological nodal-line semimetals.

In summary, we systemically combined experimental and theoretical s-QPI techniques to directly visualize the unconventional floating band type of surface state on a topological Dirac nodal line semimetal ZrSiSe, which features a non-symmorphic space group *P*4/*nmm*. Three effects, namely a rotational symmetry violation, a healing effect, and a half-missing type anomalous Umklapp process are identified as characteristic properties of a floating band. Moreover, the half-missing Umklapp process can be understood as a non-symmorphic effect, which theoretically exists in a broad class of materials whose lattices contain glide mirrors $\left( {\left. {M_z} \right|\frac{1}{2}\frac{1}{2}0} \right)$. One may potentially be able to deduce the $\left( {\left. {M_z} \right|\frac{1}{2}\frac{1}{2}0} \right)$ symmetry induced phase shift of the electron wavefunction by using the atomic manipulation technique to arrange an array of adatoms into a particular geometry^29^. Furthermore, the revealed anisotropic charge carrier scattering behavior may provide insights in the development of new nanoelectronics. Therefore, we believe our results here are of both fundamental and applicational importance.

## Methods

### Sample growth

The single crystalline ZrSiSe samples were synthetized by a standard chemical vapor transport method. A stoichiometric mixture of Zr, Si, and Se powders and the transport agent I_2_ (5 mg cm^−3^) were placed at the end of a quartz tube. The quartz tube was then evacuated, sealed and loaded into a horizontal tube held at high temperature. The occupied end, which contained the reaction powders, and empty end of the quartz tube were maintained at the high temperature 950 °C and low temperature 850 °C respectively. The temperature gradient of tube furnace was maintained for two weeks. The square and rectangular shaped ZrSiSe crystals were formed at the cold end.

### STM measurement

The STM/STS measurements were carried out in a scanning tunneling microscope (USM-1600, Unisoku) with an ultrahigh vacuum (base pressure~1 × 10^−10^ torr). The samples were cleaved in situ at 80 K and then transferred into the STM head immediately. All the measurements were performed at *T* = 4.8 K using platinum iridium tips treated with in situ electron-beam cleaning. d*I*/d*V* signals were acquired by a lock-in amplifier with modulation of 20 mV at 991 Hz. All presented Fourier transformed maps are raw data.

### DFT calculations

The first-principles calculations were based on the generalized gradient approximation^30^ (GGA) using the full-potential projector augmented-wave method^31,32^ as implemented in the VASP package^33,34^. The electronic structure of bulk ZrSiSe were calculated using a 20×20×10 Monkhorst-Pack *k*-mesh over the Brillouin zone (BZ). We also conducted the calculations of 30-layer ZrSiSe slab using a 20×20×1 Monkhorst-Pack *k*-mesh. The vacuum thickness was larger than 2 nm to ensure the separating of the slabs. The spin–orbit coupling was included. We used Zr *s*, *p*, and *d* orbitals, Si *s* and *p* orbitals, and Se *p* orbitals to construct Wannier functions without performing the procedure for maximizing localization. We combined the bulk Wannier functions and the surface part of slab Wannier functions to simulated the surface spectral weight via a semi-infinite Green’s function method.

In a ZrSiSe crystal, the glide mirror $\left( {\left. {M_z} \right|\frac{1}{2}\frac{1}{2}0} \right)$ guarantees the existence of AB sublattice and enforce the sublattice to precisely locate in the middle of a surface unit cell. The non-symmorphic effect induces a non-trivial structure factor which must be considered in a first principle simulation. In our calculation, we construct a unitary matrix U(**k**):

$${\mathrm{U}}({\mathbf{k}}) = \left( {\begin{array}{*{20}{c}} {e^{i{\mathbf{k}}r_1}} & \cdots & 0 \\ \vdots & \ddots & \vdots \\ 0 & \cdots & {e^{i{\mathbf{k}}r_n}} \end{array}} \right)$$

where **k** is the wavevector, *r*_i_ is the real space coordinates of *i*th atom in one ZrSiSe unit cell. By acting this unitary matrix with the Hamiltonian, i.e., U(**k**) H(**k**) U^+^(**k**), we are able to simulate the non-symmorphic effect. In contrast, a direct diagonalization of H(**k**) gives rise to the simulated band structure without considering non-symmorphic effect, which fails to capture the experimental results

### Simulation of s-QPI patterns using the T-matrix approach for ZrSiSe

To simulate the interference patterns, we adopted the T-matrix approach, which has been widely used in the QPI studies for the surface states on topological materials^35–38^. The retarded surface Green’s function of the system can be written as

$$G\left( {{\mathbf{k}},\omega } \right) = \left[ {E - H_{\mathrm{s}}^{{\mathrm{eff}}}\left( {\mathbf{k}} \right)} \right]^{ - 1},$$

where *E* = *ω* + *iη* with *ω* representing energy and *η* being a small broadening factor and $H_{\mathrm{s}}^{{\mathrm{eff}}}\left( {\mathbf{k}} \right)$ is an effective surface Hamiltonian calculated by semi-infinite Green’s function method.

When the interference due to the presence of a single non-magnetic impurity is considered, the Fourier transformed impurity-induced local density of states at a given scattering wavevector **q** and energy *ω* can be derived as

$$\rho _{{\mathrm{imp}}}({\mathbf{q}},\omega ) = \frac{i}{{2\pi }}{\int} {\frac{{{\mathrm{d}}^2k}}{{\left( {2\pi } \right)^2}}} g_{{\mathrm{imp}}}({\mathbf{k}},{\mathbf{q}},\omega ),$$

where the impurity-induced electronic Green’s function gives rise to *g*_imp_(**k**, **q**, *ω*) = Tr(*G*(**k**, *ω*)*T*(**k**, **k** + **q**, *ω*)*G*(**k** + **q**, *ω*)) − Tr(*G*(**k**, *ω*)*T*(**k**, **k** − **q**,*ω*)*G*(**k** − **q**,*ω*))^*^.

The T-matrix, *T*(**k**, **k**′,*ω*), can be expressed as

$$T\left( {{\mathbf{k}},{\mathbf{k}}\prime ,\omega } \right) = \left[ {1 - {\int} {\frac{{{\mathrm{d}}^2p}}{{\left( {2\pi } \right)^2}}} V_{{\mathrm{imp}}}\left( {{\mathbf{p}},{\mathbf{p}}} \right)G\left( {{\mathbf{p}},\omega } \right)} \right]^{ - 1}V_{{\mathrm{imp}}}({\mathbf{k}},{\mathbf{k}}\prime ).$$

Note that the impurity potential matrix *V*_imp_(**k**, **k**′) is induced by an impurity or a vacancy on the surface and carries k-dependent matrix elements, where **k**(**k**′) indicates out-going (in-coming) wavevector. We considered two types of vacancies on the top most surface: (1) Zr and (2) Si vacancies. We modeled a vacancy by removing all hopping terms associated with the vacancy site. The impurity potential of vacancy for *α* atom can be expressed in real space as following:

$$V_{{\mathrm{vac}}}^\alpha = - \mathop {\sum }\limits_{i \vee j \in \alpha } c_i^\dagger H_{ij}c_j,$$

where *H*_*ij*_ denotes the hopping amplitude between two orbitals *i* and *j*. The summation over any one of *i* or *j* basis belonging to *α* atom site is claimed to ensure that no interactions between the vacancy site *α* and the surroundings. *V*_imp_(**k**, **k**′) was obtained from a Fourier transform. Our final results are obtained by extracting the signal of Se orbitals on the topmost surface from *ρ*_imp_(**q**, *ω*) by assuming the tunneling currents are only from atoms on the topmost surface in scanning tunneling microscopy measurement.

## Electronic supplementary material

Supplementary Information

## Data Availability

All relevant data that support the findings of this study are available from the corresponding author upon reasonable request.
