# Supplementary material for: Quasiparticle interference and nonsymmorphic effect on a floating band surface state of ZrSiSe
Source: Nat Commun. 2018 Oct 8;9:4153. doi: 10.1038/s41467-018-06661-9 (PMC6175950; doi:10.1038/s41467-018-06661-9)
Supplement: Supplementary file 1 — Supplementary Information [file 41467_2018_6661_MOESM1_ESM.pdf]

Supplementary Figures:

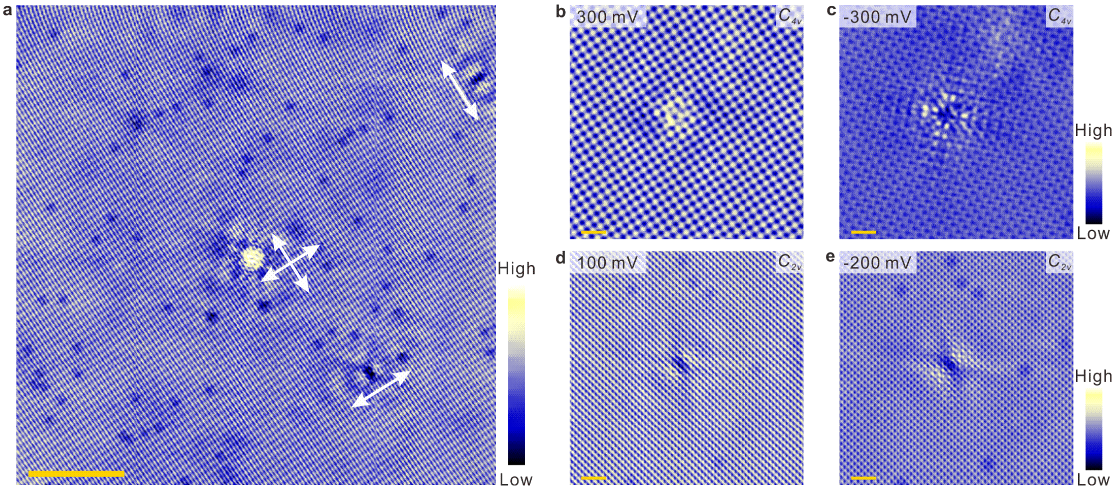

Supplementary Figure 1| STM images of ZrSiSe morphology and defects. a, STM image showing a large scale atomically flat ZrSiSe (001) surface. Arrows (cross) mark two C_{2v} (C_{4v}) defects. Scale bar stands for 10nm. **b** and **c**, positive and negative biased STM images on a C_{4v} symmetric defect revealing its empty and occupied states respectively. **d** and **e**, positive and negative biased STM images on a C_{2v} symmetric defect. Scale bars in **b** to **e** stand for 1nm.

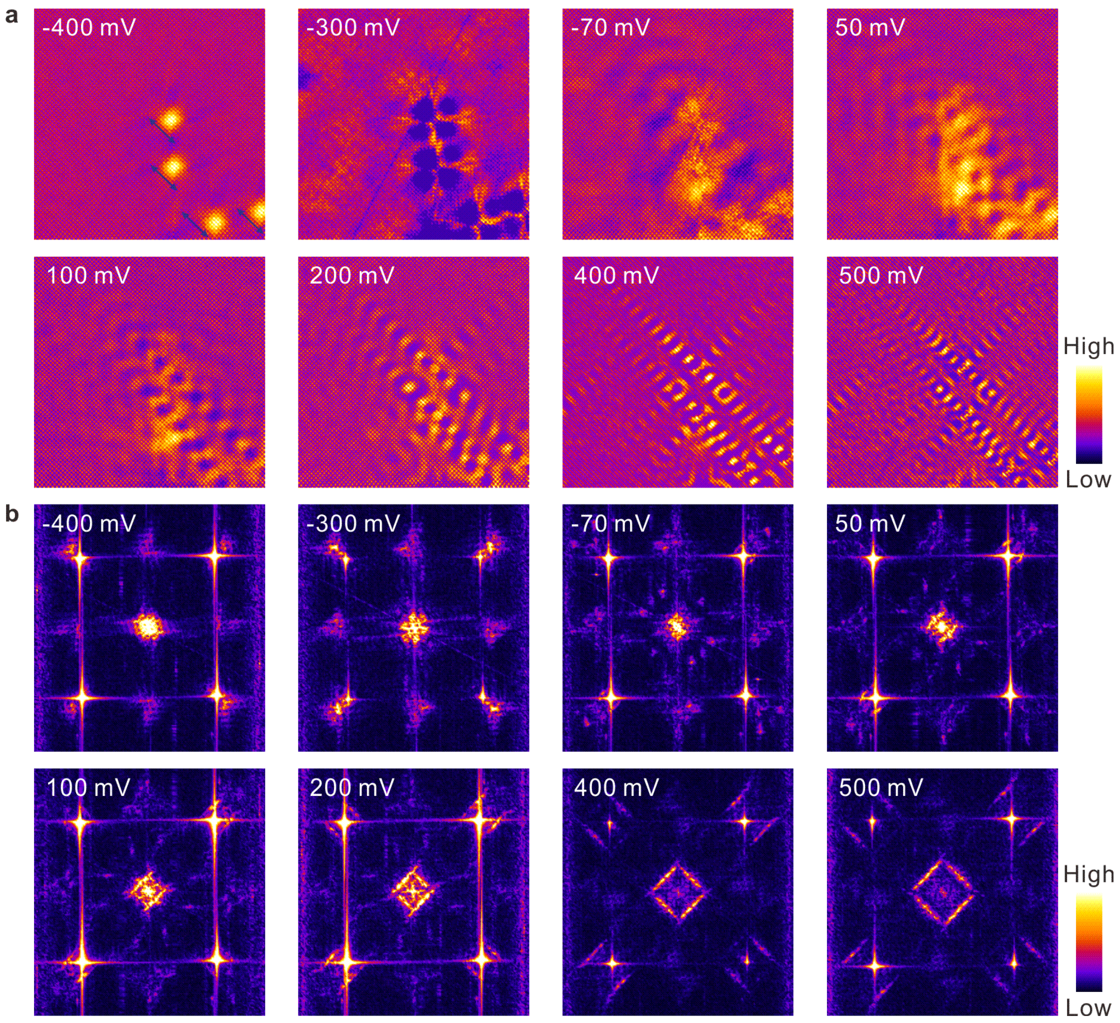

Supplementary Figure 2| Quasiparticle interference (QPI) on an area containing four C_{2v} defects unidirectionally orientated. a, spatially resolved dI/dV conductance maps at indicated voltages. Arrows mark the main wave-propagating directions. The four defects orientate in same direction. Multiple unidirectional defects extensively enhance the QPI intensity. **b,** Corresponding Fourier transform (FT) of each map in panel **a**.

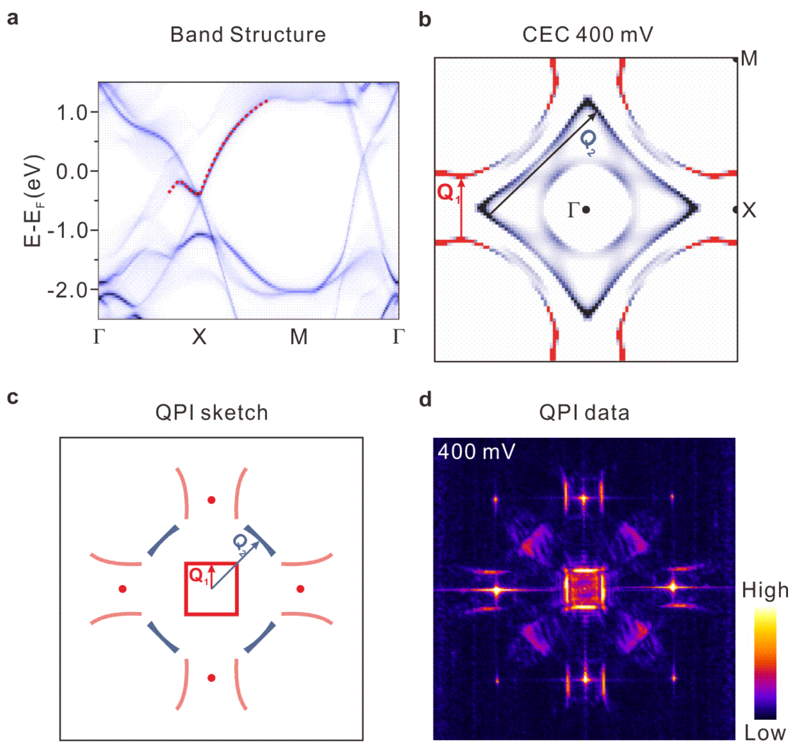

Supplementary Figure 3| Schematic of surface floating band and relevant QPI signal on ZrSiSe(001). a, Calculated surface band structure of ZrSiSe(001). The floating band state is highlighted in red. **b**, Calculated constant energy contour (CEC) at 400 meV. $\mathbf{Q_1}$ stands for the scattering between two floating band pockets while $\mathbf{Q_2}$ is an intrapocket scattering within the pocket around Γ that is generated by other surface states. Please note that the $\mathbf{Q_2}$ in this figure has different meaning than the $\mathbf{Q_2}$ in Figs. 4 **a** and **b** in main text. **c**, A sketch shows the QPI pattern expected for the CEC in **b**. The red square arises from the scattering between floating bands. The pink double-arcs denote corresponding Umklapp processes. **d**, a measured single-defect-induced QPI (s-QPI) map (same as the one of 400 meV in Fig. 3 **b**), which indeed possesses all highlighted features in **c.**

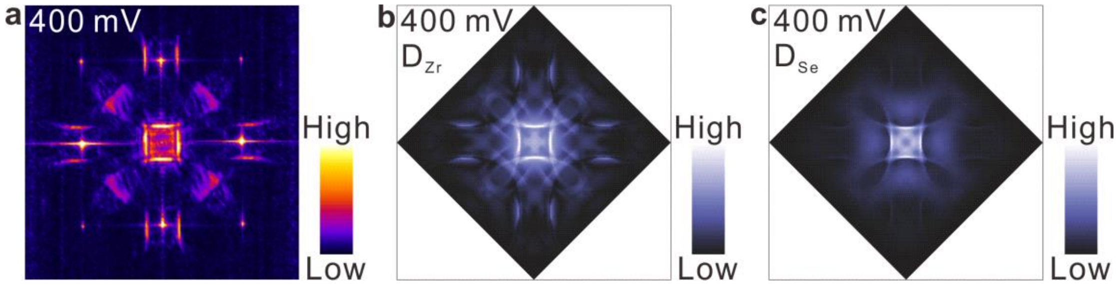

Supplementary Figure 4| Identification of chemical nature of a C_{4v} defect. a, s-QPI map of an isolated defect with C_{4v} symmetry at 400 meV (same as the one of 400 meV in Fig. 3 **b**). **b** and **c,** Theoretically simulated s-QPI patterns of a single Zr and Se vacancy at 400 meV, respectively. The experimental pattern is in better accordance with the calculated result based on a Zr defect.

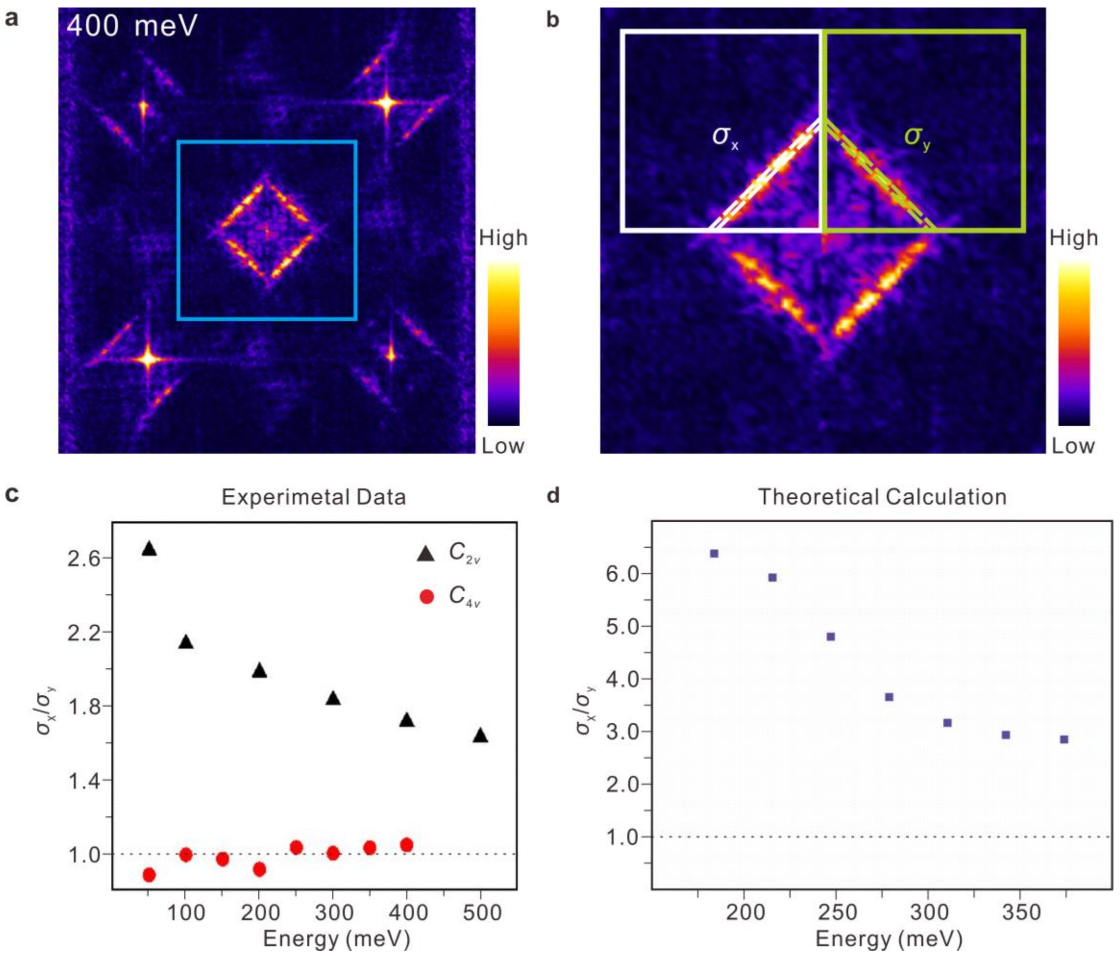

Supplementary Figure 5| Identification of C_{2v} and C_{4v} symmetries from intensity ratio of QPI signals. a, experimental QPI map of uni-directional Si defects with C_{2v} symmetry at 400 meV (same as the one of 400 meV in Fig. S2 **b**). **b**, zoom-in image of the feature inside the square of **a**. We define the average value of each point along the two dashed lines that possess the strongest signal in the white and green square as σ_x and σ_y. **c**, the measured energy dependent values of σ_x/σ_y. The σ_x/σ_y values from Si (C_{2v}) and Zr (C_{4v}) defects are represented by red balls and black triangles, respectively. For C_{4v} symmetric defects (red), σ_x/σ_y values are all around 1 at different energies. However, the σ_x/σ_y values from C_{2v} symmetric defects (black) are all larger than 1 and gradually change to be close to 1 with elevated energies. It clearly demonstrates that the four-folds rotational symmetry is broken at Fermi level but is gradually restored at high energy around Si defect. **d**, the theoretical σ_x/σ_y values from simulated C_{2v} symmetric s-QPI patterns. It clearly reproduces the symmetry broken and healing effect from Si defect.

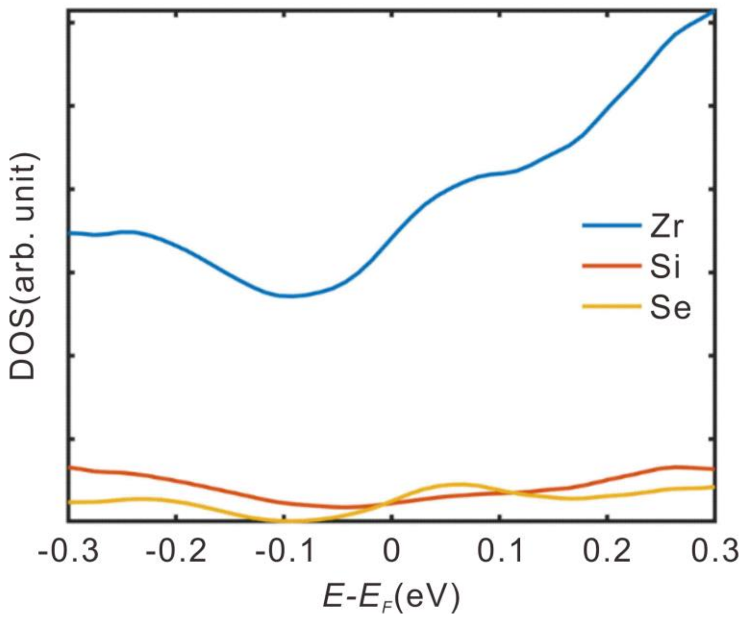

Supplementary Figure 6| Partial Density of state (DOS) from different atoms. Zr, Si and Se derived signals are represented by blue, red and yellow, respectively. Note that above the Fermi level, the intensity of Zr induced partial DOS clearly increases, while the Si induced partial DOS remains rather constant. The rapidly increasing Zr DOS intensity contributes more significantly to the s-QPI around a Si defect, and thus leads to the enhancement of C_{4v} feature recovery when the bias is increased.

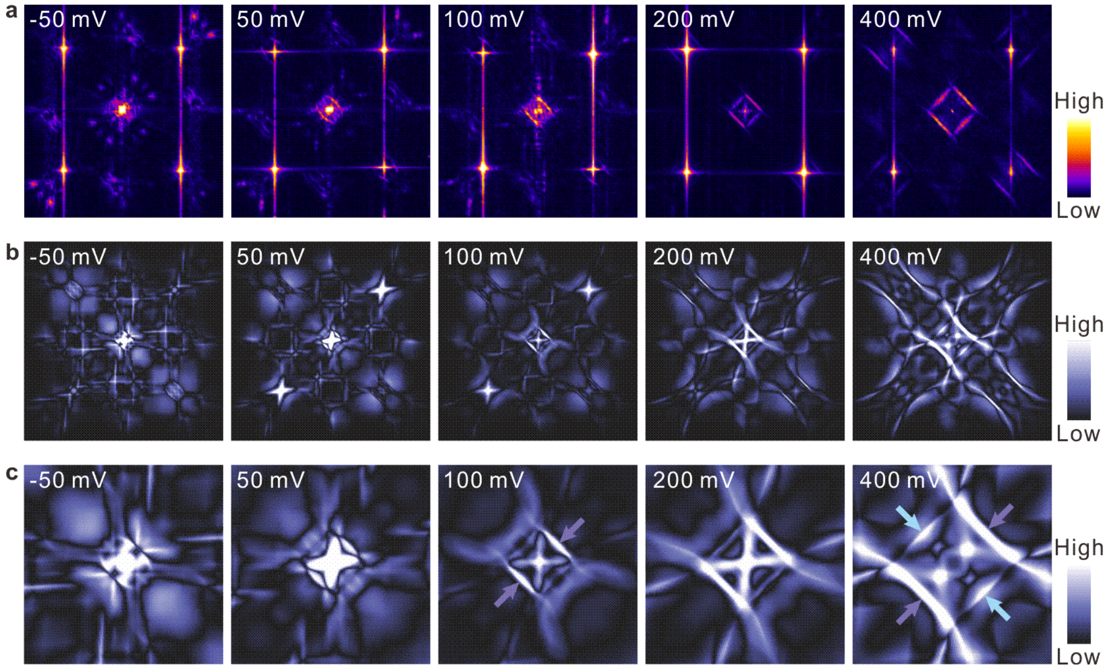

Supplementary Figure 7| Comparison of the FT-d*I*/d*V* images of a single Si defect with predicted s-QPI patterns. **a**, s-QPI maps (same as Fig. 2 **b**). **b**, corresponding theoretically simulated s-QPI patterns based on a single Si vacancy, which reasonably reproduce the features shown in **a**. **c**, zoom-in images of the central QPI pockets in **b**, which clearly show the C_{4v} symmetry is gradually restored at high biases. For example, in the image at 100meV, the dominant QPI feature is the bright double-arcs as indicated by the purple arrows. However, in the image at 400 meV, the QPI pocket grows in size and evolves into a nearly square shape.

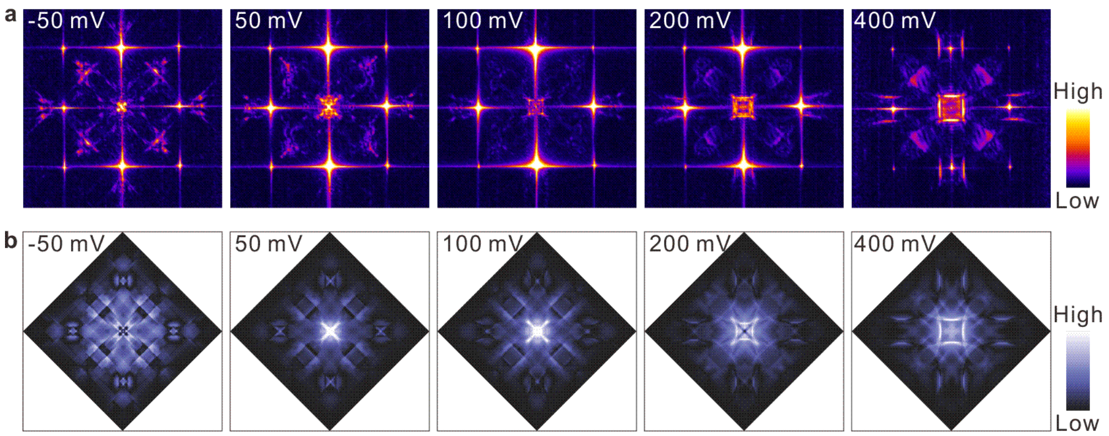

Supplementary Figure 8| Comparison of the FT-d*I*/d*V* images of a single Zr defect with predicted s-QPI patterns. a, s-QPI maps (same as Fig. 3 **b**). **b**, corresponding theoretically simulated s-QPI patterns based on a single Zr vacancy. The obtained data and the calculations are in agreement over a wide energy range.

Supplementary Discussion

Supplementary Figure 7 shows s-QPI patterns of single Si vacancy on topmost surface at various energies. A Si vacancy gives rise to patterns with C_{2v} symmetry from *p*-orbital scattering channels. The crucial signal is double-arc-shaped features in the center and near two Bragg points. When the energy increases, the QPI signal intensity becomes stronger while the distance between two arcs increases, additionally the QPI patterns evolves into a C_{4v} like shape. The healing effect is mainly caused by surface states scattering through *p*-orbit channels.

Supplementary Figure 8 shows s-QPI patterns of single Zr vacancy on topmost surface at various energies. A Zr vacancy produces patterns with C_{4v} symmetry mainly contributed from *d*-orbital scattering channels. The square-like shape at center is enhanced when energy increases. Notably, the double arc-shaped features rather than square appear near the Bragg points. This is the anomalous half missing Umklapp process, which is a characteristic of the *P*4/*nmm* nonsymmorphic symmetry.
